# A Case of Autoimmune Pulmonary Alveolar Proteinosis With Predominantly Peripheral Opacities Diagnosed by Transbronchial Lung Biopsy

**DOI:** 10.7759/cureus.54261

**Published:** 2024-02-15

**Authors:** Takeshi Imakura, Soji Kakiuchi, Mami Inayama, Ayaka Mori, Takashi Haku

**Affiliations:** 1 Department of Respiratory Medicine, Tokushima Prefectural Central Hospital, Tokushima, JPN

**Keywords:** anti-gm-csf antibody, subpleural, ground-glass shadow, transbronchial lung biopsy, autoimmune pulmonary alveolar proteinosis

## Abstract

Although pulmonary alveolar proteinosis (PAP) showed various shadows, its shadows are usually distributed predominantly in the central lung area. We report a case of autoimmune PAP with localized subpleural ground-glass shadows in the bilateral upper lobes, which was diagnosed based on transbronchial lung biopsy (TBLB) specimen findings and anti-granulocyte macrophage colony PAP stimulating factor antibody positivity. PAP should be listed as a differential diagnosis for subpleural shadows. If subpleural shadows are observed, TBLB should be performed aggressively, and anti-granulocyte macrophage colony-stimulating factor (anti-GM-CSF) antibodies should be submitted.

## Introduction

Pulmonary alveolar proteinosis (PAP) is a rare disease characterized by progressive respiratory failure due to excessive surfactant accumulation in alveoli and respiratory bronchioles [1]. PAP can be classified into three categories according to its etiology: (1) autoimmune pulmonary alveolar proteinosis (APAP), (2) secondary pulmonary alveolar proteinosis (SPAP), and (3) congenital/hereditary pulmonary alveolar proteinosis (CPAP/HPAP) [2]. Ninety to ninety-five percent of adult PAP cases are APAP, while SPAP and CPAP/HPAP account for a small percentage of cases [2]. Computed tomography (CT) of the PAP showed ground-glass opacities, interlobular septal thickening, intralobular reticular shadows, and a crazy-paving pattern with overlapping shadows. Shadows are generally predominantly distributed in the central lung area near the hilar region [3]. If the shadows of PAP appear in the peripheral lung area, central opacities are also mostly present. Shadows of PAP rarely appear in the peripheral lung area [3]. We report a case of APAP with localized subpleural shadows in the bilateral upper lobes, which was diagnosed based on TBLB specimen findings and anti-GM-CSF antibody positivity.

## Case presentation

**Figure 1:**
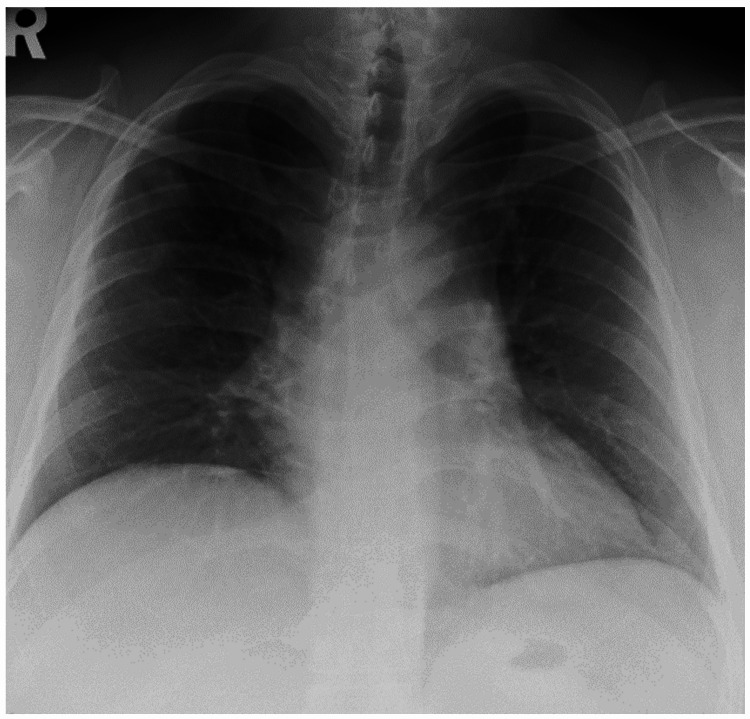
A chest radiograph obtained in January 2020 showed patchy shadows in the right upper lung field.

**Figure 2:**
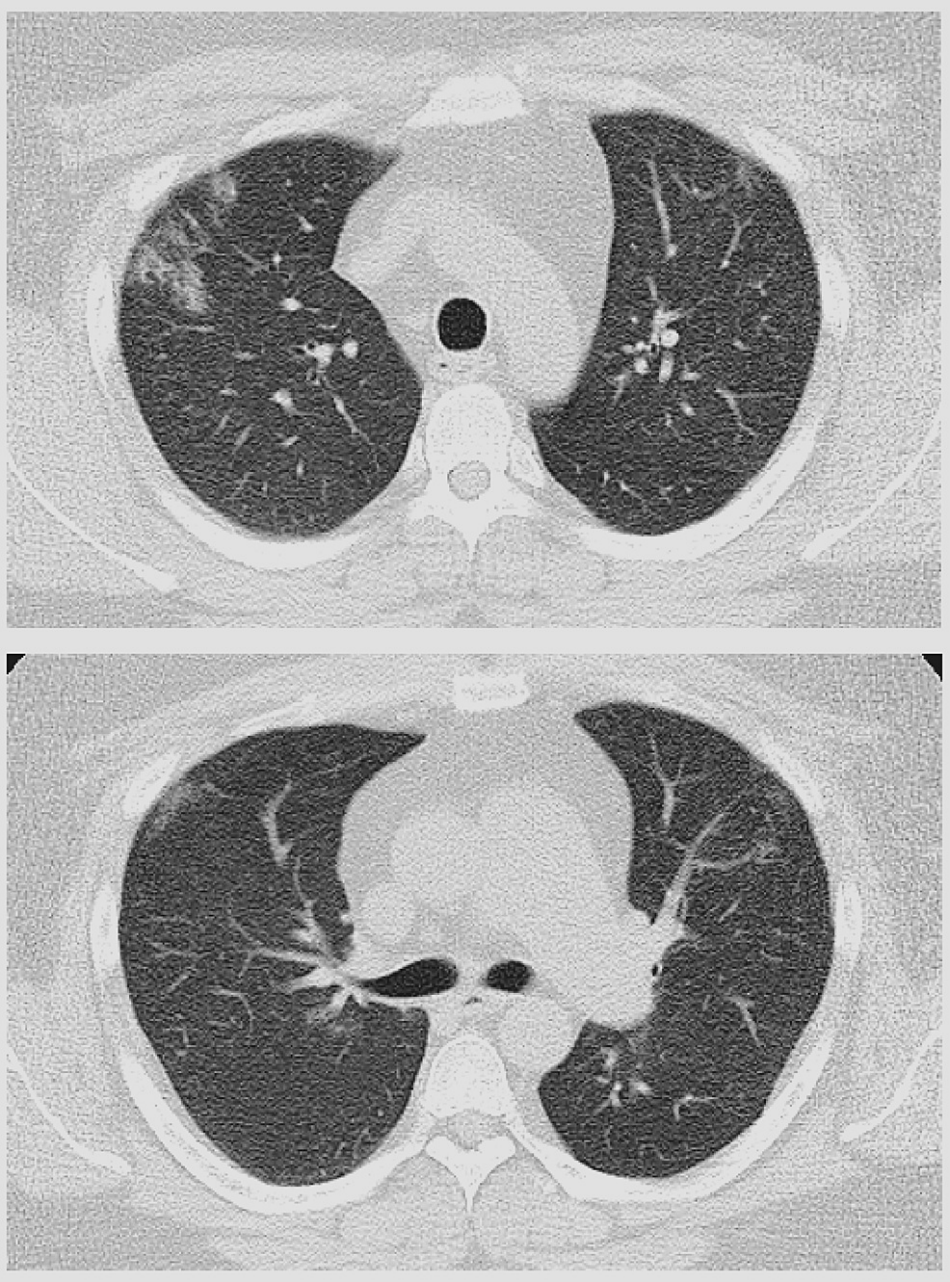
CT in January 2020 showed ground-glass opacities and infiltrative shadows in the subpleural area of the right upper lobe and ground-glass shadows in the left upper lobe. CT, computed tomography

**Figure 3:**
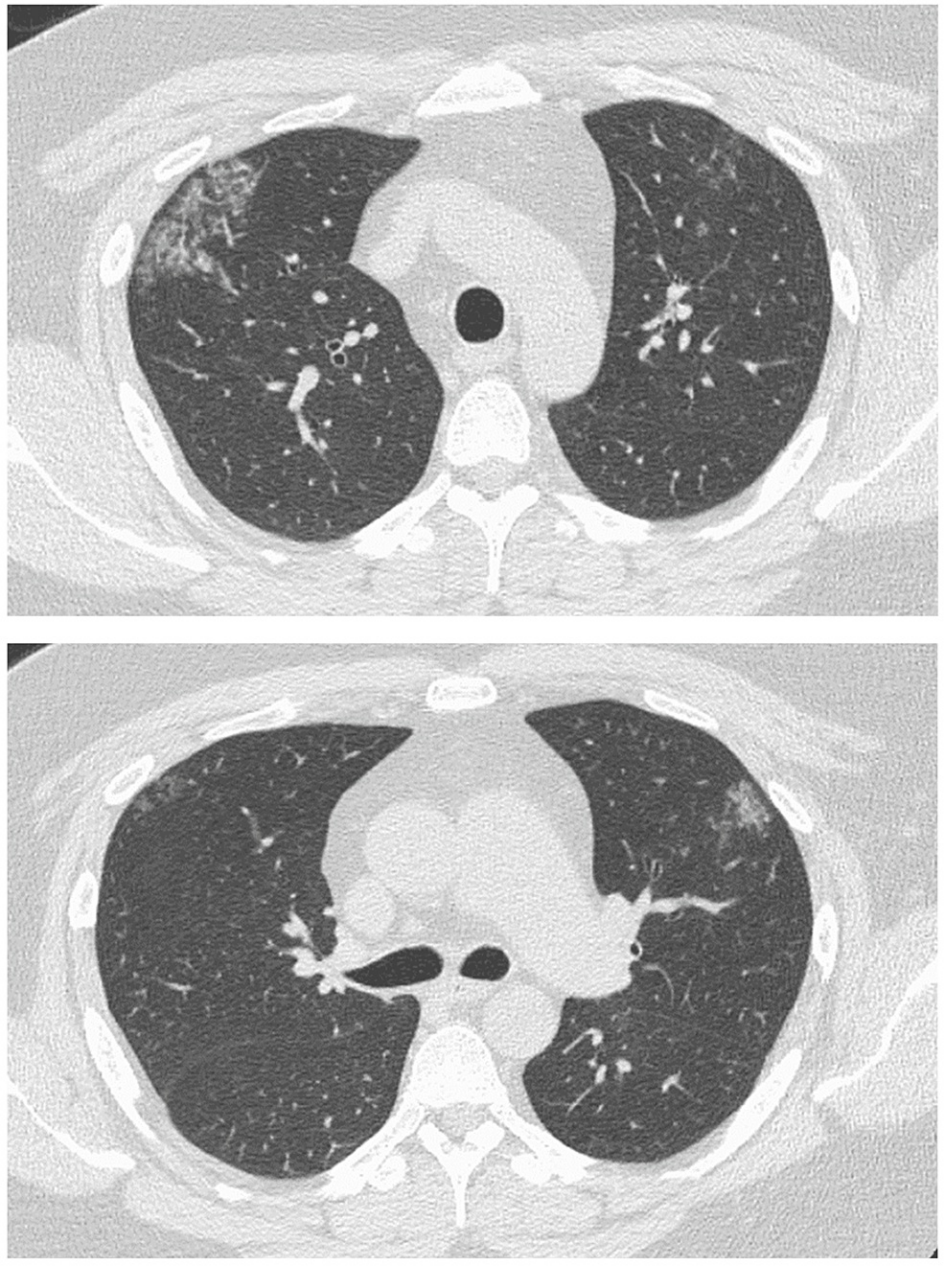
CT in January 2022 showed ground-glass opacities and infiltrative shadows in the subpleural area of both upper lobes, which were exacerbated in comparison to January 2020. CT, computed tomography

**Figure 4:**
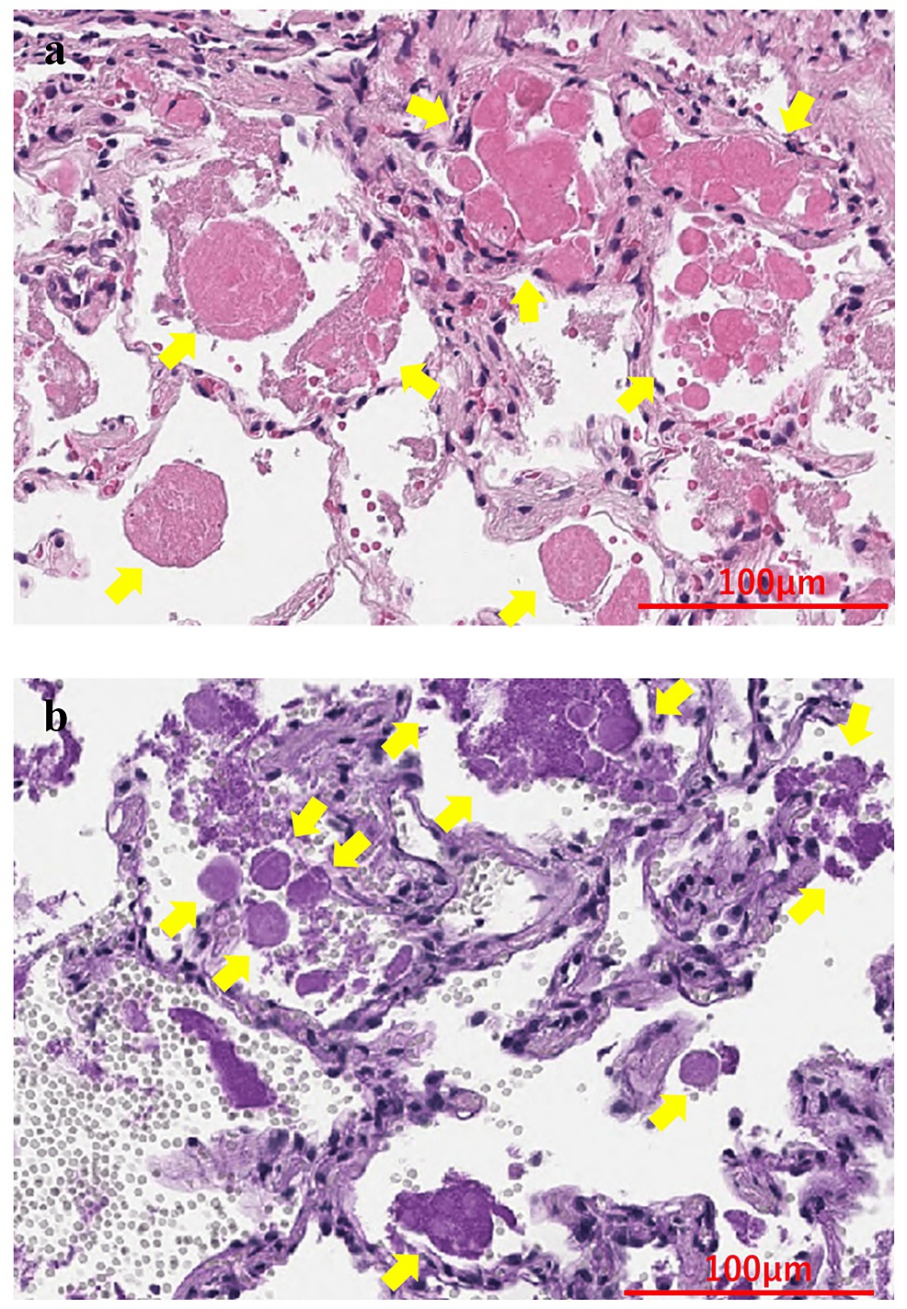
Histological findings of the TBLB specimen. A histological examination was performed with Hematoxylin and Eosin (a) and PAS staining (b). The biopsy specimens contained eosinophilic bodies that were strongly stained with PAS. Yellow arrows indicate eosinophilic bodies. A scale bar is shown in each figure. TBLB, transbronchial lung biopsy; PAS, periodic acid-Schiff

**Figure 5:**
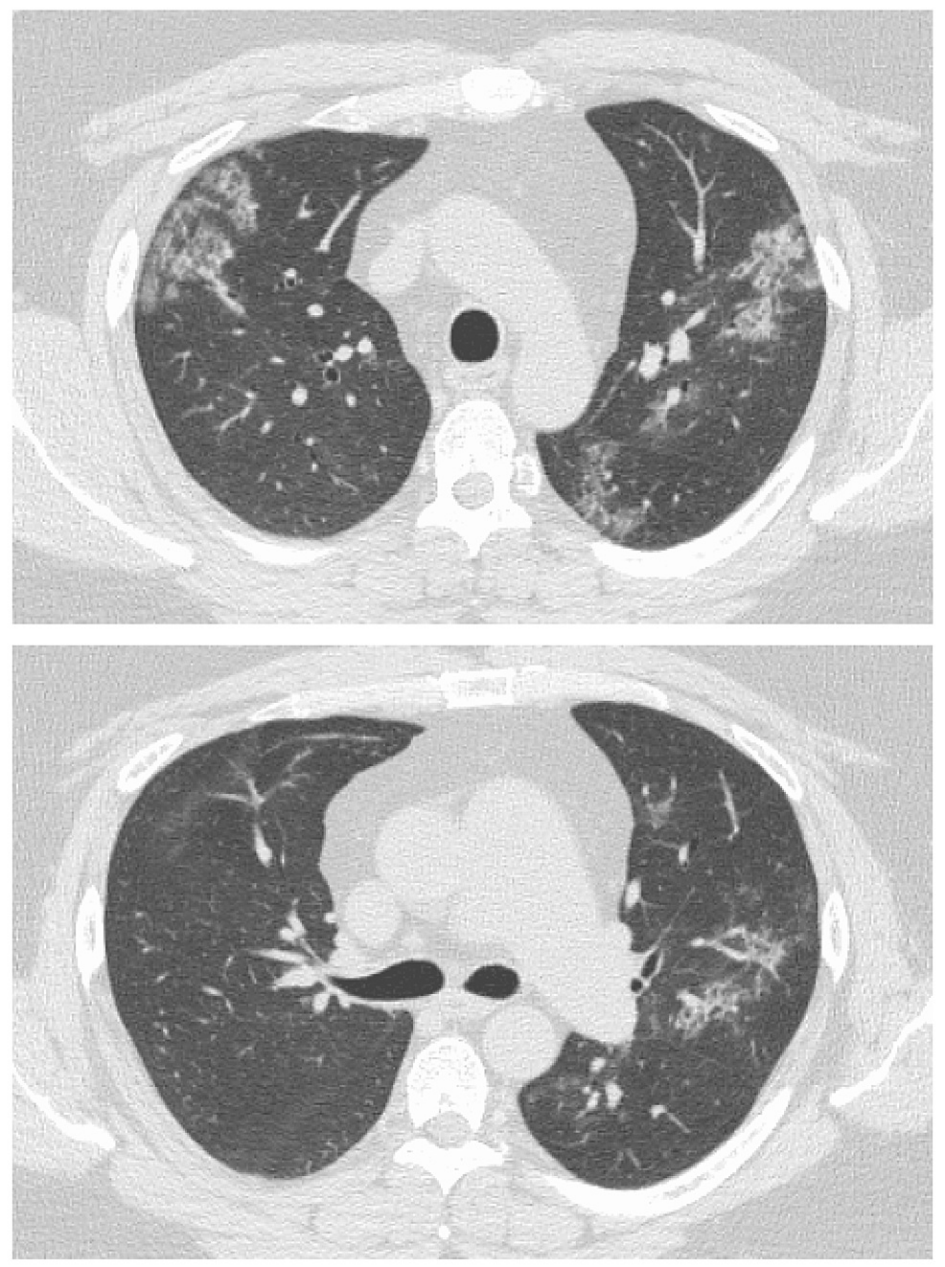
CT in July 2023 showed the appearance of central-side opacities. CT, computed tomography

A 38-year-old man was a non-smoker with a history of bronchial asthma and type 2 diabetes mellitus. In January 2020, a chest radiograph taken during a medical checkup showed patchy shadows in the right upper lung field (Figure 1). Chest CT showed ground-glass shadows and infiltrative shadows in the subpleural area of the right upper lobe and ground-glass shadows in the left upper lobe (Figure 2). As there were no symptoms, the patient was not closely examined. However, a chest CT performed during a medical checkup in December 2021 showed enhanced ground-glass shadows and infiltrative shadows in both upper lobes. Therefore, the patient was referred to our hospital in January 2022. He was asymptomatic, and his initial vital signs were as follows: blood pressure, 148/98 mmHg; body temperature, 36.7°C; percutaneous oxygen saturation (SpO_2_), 98%. Lung sounds were normal on auscultation. There was no evidence of arthralgia, skin rash, or other symptoms suggestive of collagen disease. Other physical examination findings were also normal. Blood tests showed the elevation of lactate dehydrogenase (LDH), Krebs von den Lungen-6 (KL-6), and carcinoembryonic antigen (CEA) (Table 1). Peripheral smears showed no abnormalities. Chest radiography showed patchy shadows in the right upper lung field, and a chest CT scan showed ground-glass shadows and infiltrative shadows in the subpleural area of both upper lobes, which were exacerbated in comparison to January 2020 (Figure 3). Considering the possibility of malignancy or organizing pneumonia, bronchoscopy with bronchoalveolar lavage (BAL) and transbronchial lung biopsy (TBLB) were performed in February 2022. BAL was performed on the right B5a, and 101/150 mL was collected. The bronchoalveolar lavage fluid (BALF) showed a mild milky appearance. The total number of cells increased, and the lymphocyte fraction increased to 27.5%, indicating the possibility of interstitial pneumonia responsive to corticosteroids (Table 2). TBLB was performed at the B3aii and B4aii positions. TBLB specimens showed that the alveoli were filled with periodic acid-Schiff (PAS)-positive eosinophilic amorphous materials, and PAP was strongly suspected (Figure 4). The serum level of anti-granulocyte macrophage colony-stimulating factor (anti-GM-CSF) antibody increased to 33.6 U/mL (a reference range was 0-1.7 U/mL). On the basis of these findings, the patient was diagnosed with APAP. Because he had no subjective symptoms, he had not received any treatment since then. However, in July 2023, central-side opacities appeared on chest CT, he developed dyspnea on exertion, and the modified Medical Research Council Dyspnea Scale was 1 (Figure 5). Currently, there are no effective drugs available in Japan for PAP. Therefore, we would follow him without any treatments. However, when the imaging findings and subjective symptoms worsen further, we intend to consider performing BAL.

**Table 1:** The serum test results at the initial visit to our department. WBC, white blood cell; Neut, neutrophil; Eo, eosinophil; Baso, basophil; Mono, monocyte; Lymp, lymphocyte; Hb, hemoglobin; Plt, platelet count; AST, aspartate aminotransferase; ALT, alanine aminotransferase; LDH, lactate dehydrogenase; BUN, blood urea nitrogen; Cre, creatinine; CRP, C-reactive protein; KL-6, krebs von den lugen-6; CEA, carcinoembryonic antigen; CYFRA, cytokeratin 19 fragment; Pro-GRP, pro-gastrin releasing peptide; T-SPOT, T-SPOT®-TB; MAC, Mycobacterium avium complex; βD-glucan, beta-d-glucan; ANA, antinuclear antibody; IgG, immunoglobulin G; IgG4, immunoglobulin G4

Parameters	Results	Reference range
WBC	8200 /µL	3500-9100/µL
Neut	56.10%	44.0-72.0%
Eo	6.80%	0.0-10.0%
Baso	2.20%	0.0-3.0%
Mono	5.20%	0.0-12.0%
Lymp	29.70%	18.0-59.0%
Hb	16.8 g/dL	11.3-15.2 g/dL
Plt	28.3×10⁴ /µL	13-36.9×10⁴ /µL
AST	23 U/L	10-35 U/L
ALT	28 U/L	5-40 U/L
LDH	236 U/L	124-222 U/L
BUN	16.3 mg/dL	7-20 mg/dL
Cre	0.88 mg/dL	0.4-0.9 mg/dL
Na	144.1 mEq/dL	135-146 mEq/dL
K	4.62 mEq/dL	3.5-4.8 mEq/dL
KL-6	968 U/mL	50-500 U/mL
CRP	0.06 mg/dL	0-0.5 mg/dL
CEA	5.9 ng/mL	0-5 ng/mL
CYFRA	1.1 ng/mL	0-3.5 ng/mL
Pro-GRP	44.1 pg/mL	0-31 pg/mL
T-SPOT	(-)	-
panel A	0 spot	0-7 spot
panel B	0 spot	0-7 spot
anti-MAC antibody	(-)	-
βD-Glucan	9 pg/mL	0-20 pg/mL
ANA	<40 times	0-40 times
IgG	1461 mg/dL	870-1700 mg/dL
IgG4	107 mg/dL	11-121 mg/dL

**Table 2:** The results of BALF. *CD4+/CD8+ T-lymphocyte ratio. BALF, bronchoalveolar lavage fluid

Parameters	Results	Reference range
Recovery rate	67.3%	-
	101 mL/150 mL	
Total cell counts	2.0×10^5^/mL	0.7-2.0×10^5^/mL
Cell fractionation		
Alveolar macrophages	71.5%	80-90%
Lymphocytes	27.5%	10-15%
Neutrophils	1.0%	0-3%
Eosinophils	0.0%	0-1%
CD4/8 ratio*	1.8	1.0-3.0
Cytodiagnosis	Class Ⅱ	-
BALF cultures	Negative	-
Appearance	Mild milky	-

## Discussion

APAP is the most common form of PAP and accounts for approximately 90% of all cases. In a Japanese survey of 223 reported cases between 1999 and 2006, the incidence was 0.24 per million, and the prevalence was 2.04 per million [4]. In the same survey, the incidence was 0.49 per million and the prevalence was 6.2 per million in Niigata Prefecture, Japan [4]. On the other hand, in a survey conducted between 2006 and 2016 in Niigata Prefecture, Japan, the incidence was 1.65 per million and the prevalence was 26.6 per million, which amounted to a considerable increase in comparison to previous surveys [5]. This may be due to the fact that anti-GM-CSF antibody measurements have become widespread in recent years, and potential cases that had not been diagnosed in the past have actually been diagnosed with APAP. Thus, although APAP is a rare disease, its incidence and prevalence may be higher than previously assumed, and it should be considered as a differential diagnosis for abnormal chest shadows.

Usually, PAP shadows are distributed predominantly in the central lung area [3]. However, although rare, there have been case reports (the present case and 13 previously reported cases) of PAP in which shadows were predominantly distributed in the peripheral lung area (Table 3) [6-17]. As in the present case, there were 11 cases with no dyspnea and six cases in which the BALF had a milky appearance. A surgical lung biopsy was performed in five cases, and TBLB was performed in 10 cases. The mean lymphocyte fraction of BALF in the six cases for which data were available was 25%. Serum anti-GM-CSF antibodies were positive in nine patients. There are two possible explanations as to why any shadows are distributed in the peripheral areas. One is that subpleural shadows show the early stage of PAP and are a prelude to the future appearance of central-side opacities [6,9]. The other is that subpleural shadows are residual lesions after the central opacities have spontaneously disappeared [6,7]. In this case, central ground-glass opacities appeared during the follow-up, which may support the hypothesis that peripheral opacities represented early PAP. However, the number of reported cases remains small, and further case accumulation and investigation are required.

**Table 3:** Summary of PAP patients with peripheral predominant opacities reported in the literature. PAP, pulmonary alveolar proteinosis; BALF, bronchoalveolar lavage fluid; anti-GM-CSF, anti-granulocyte macrophage colony-stimulating factor; TBLB, transbronchial lung biopsy; BAL, bronchoalveolar lavage; TBLB, transbronchial lung biopsy; SLB, surgical lung biopsy

Case	Country	Age	Sex	Smoking history	Symptoms	Appearance of BALF	Diagnostic procedure	Lymphocyte fractionation of BALF	Anti-GM-CSF antibodies	Reported year
1. Inui et al., 1999 [6]	Japan	39	F	Never	No	Transparent	BAL, TBLB, SLB	11.50%	None	1999
2. Mita et al., 2003 [7]	Japan	38	F	Unknown	No	White	BAL, TBLB	Unknown	None	2003
3. Sugimoto et al., 2006 [8]	Japan	55	F	Never	No	White	BAL, TBLB	20%	Positive (BALF)	2006
4. Mohri et al., 2007 [9]	Japan	32	F	Never	No	White	BAL, TBLB	25.60%	Positive (BALF, serum)	2007
5. Taniguchi et al., 2008 [10]	Japan	58	F	Never	No	-	SLB	-	Positive (serum)	2008
6. Yamasaki et al., 2008 [11]	Japan	56	F	Never	No	Transparent	BAL, TBLB, SLB	Unknown	Positive (serum)	2008
7. Toyama et al., 2008 [12]	Japan	45	M	Never	No	Transparent	BAL, TBLB, SLB	Unknown	Positive (serum)	2008
8. Haga et al., 2009 [13]	Japan	65	F	Never	White		BAL, TBLB	Unknown	Positive (serum)	2009
9. Sunadome et al., 2010 [14]	Japan	57	M	Never	No	-	SLB	-	-	2010
10. Satoh et al., 2012 [15]	Japan	55	F	Never	Dyspnea	White	BAL, TBLB	15%	Positive (serum)	2012
11. Sugino et al., 2019 [16]	Japan	41	M	Never	No	Transparent	BAL, TBLB	50.60%	Positive (serum)	2019
12. Fujii et al., 2022 [17]	Japan	58	F	Never	Dyspnea	Transparent	BAL, TBLB	Unknown	Positive (serum)	2022
13. Present case	Japan	38	M	Never	No	White	BAL, TBLB	27.50%	Positive (serum)	2024

Usually, the lymphocyte fraction of BALF increases in PAP, and Azuma et al. reported a median lymphocyte fraction of 42% in 78 cases of PAP [18]. The percentage of lymphocyte fraction in BALF was also increased in PAP with peripheral-predominant opacities, as in the present case (Table 3). Because of the presence of ground-glass shadows and infiltrative shadows, an increased lymphocyte fraction in BALF, and elevated serum markers, such as KL-6 and SP-D, PAP could have been misdiagnosed as interstitial pneumonia, in particular nonspecific interstitial pneumonia or organizing pneumonia [19]. Particularly in the case of PAP with peripheral-predominant opacities, we believe that the possibility of misdiagnosing interstitial pneumonia and initiating immunosuppressive therapy with corticosteroids or other immunosuppressive drugs is even greater due to the atypical imaging findings. In fact, in some previous reports of PAP with peripheral predominant opacities, patients were initially diagnosed with interstitial pneumonia, and immunosuppressive treatments including corticosteroids were initiated before they were diagnosed with PAP [15,17]. However, it has been reported that corticosteroid treatment is not only ineffective for PAP but also worsens the disease [20]. This is thought to be due to the fact that PAP is more likely to be complicated by infection because of the reduced function of alveolar macrophages and neutrophils, and the administration of corticosteroids may further increase the risk of infection [20]. These findings suggest that distinguishing between PAP with interstitial pneumonia is important. When an abnormal chest image is observed, as in the present case, performing TBLB and submitting anti-GM-CSF antibodies should be aggressively considered based on the possibility of PAP.

## Conclusions

We reported a case of APAP with predominantly peripheral opacities. Although APAP is a rare disease, its incidence and prevalence may be higher than previously assumed, and it should be considered as a differential diagnosis for abnormal chest shadows. Notably, while PAP shadows are usually distributed predominantly in the central lung area, some cases showed any shadows predominantly distributed in the peripheral lung area. If an abnormal chest image is observed, TBLB and anti-GM-CSF antibody tests should be aggressively performed in order to differentiate PAP from other diseases. It will be particularly important to differentiate PAP from interstitial pneumonia, as the treatment methods are very different.
